# An overview of modern machine learning methods for effect measure modification analyses in high-dimensional settings

**DOI:** 10.1016/j.ssmph.2025.101764

**Published:** 2025-02-13

**Authors:** Michael Cheung, Anna Dimitrova, Tarik Benmarhnia

**Affiliations:** Scripps Institution of Oceanography, University of California, San Diego, CA, USA

**Keywords:** Effect measure modification, Heterogeneity, Machine learning, Generalized random forest, Bayesian additive regression trees, Bayesian causal forest, Metalearner

## Abstract

A primary concern of public health researchers involves identifying and quantifying heterogeneous exposure effects across population subgroups. Understanding the magnitude and direction of these effects on a given scale provides researchers the ability to recommend policy prescriptions and assess the external validity of findings. Traditional methods for effect measure modification analyses require manual model specification that is often impractical or not feasible to conduct in high-dimensional settings. Recent developments in machine learning aim to solve this issue by utilizing data-driven approaches to estimate heterogeneous exposure effects. However, these methods do not directly identify effect modifiers and estimate corresponding subgroup effects. Consequently, additional analysis techniques are required to use these methods in the context of effect measure modification analyses. While no data-driven method or technique can identify effect modifiers and domain expertise is still required, they may serve an important role in the discovery of vulnerable subgroups when prior knowledge is not available. We summarize and provide the intuition behind these machine learning methods and discuss how they may be employed for effect measure modification analyses to serve as a reference for public health researchers. We discuss their implementation in R with annotated syntax and demonstrate their application by assessing the heterogeneous effects of drought on stunting among children in the Demographic and Health survey data set as a case study.

## Highlights

• We summarize common machine learning methods for heterogeneous effect estimation.
• We provide a practical guide to use these methods in effect modification analyses.
• We demonstrate their application in R with a case study.

## 1 Introduction

Effect measure modification (EMM) (or treatment effect heterogeneity) is present when there are differences in an exposure-outcome relationship across subgroups in a population and constitutes an important consideration for public health researchers (VanderWeele, 2009). Said differently, we say that M is a modifier of the effect of A on Y when the average treatment effect of A on Y varies across levels of M. Since the average treatment effect of A on Y can be measured using various effect measures on either multiplicative or additive scales, the presence of effect modification depends on the effect measure being used (VanderWeele & Knol, 2014). In this context, we can define effect modifiers as variables that will contribute to effect heterogeneity.

Understanding the effect of an exposure on a given outcome within population subgroups is important for several reasons. First, it can guide intervention prioritization for those who will benefit more (depending on the scale of interest) from the treatment (VanderWeele & Knol, 2014). EMM analyses can also determine if an exposure is harmful or beneficial to a subgroup when the population level effect is zero or trends in the opposite direction (Toward Precision Medicine, 2011; VanderWeele & Knol, 2014). Discovery of this effect modification advances the understanding of a potentially complex relationship between an exposure and outcome. Furthermore, quantifying EMM is critical to external validity applications including transportability and generalizability analyses (Lesko et al., 2017). Indeed, the main reason for which effect estimates in a given study population may not be generalizable (to the target population) or transportable to another population is because of a differential distribution of effect modifiers.

Traditional methods for EMM analyses include two approaches: i) conducting stratified analyses coupled with a heterogeneity test; ii) including an interaction term in a multivariable model. The first approach consists of running separate models on subgroups and comparing subgroup treatment effects using a hypothesis test such as Cochran's Q test (Kaufman & MacLehose, 2013). The second approach involves parametric regression modeling in which heterogeneity is assessed by an interaction term between the exposure variable and effect modifier(s). While this technique estimates EMM under the potential outcomes framework, we note that the concepts of EMM and causal interaction fundamentally differ. The term “interaction” can refer to either a statistical interaction such as effect modification or causal interaction. However, when mobilizing the concept of causal interaction, we aim at manipulating both the exposure of interest A and a third variable of interest M, hypothesizing a joint intervention. This requires that the identification assumptions hold for both A and M, which is not the case when mobilizing the concept of effect modification.

Several problems exist with traditional methods. These approaches require manual specification of effect modifiers and confounders, which is often burdensome or simply not feasible for high-dimensional and nonlinear relationships. Assuming simple relationships between these variables is often naïve with real data and repeated modeling to detect heterogeneity induces multiple comparisons (Greenland, 2008). Moreover, distributional assumptions may not hold with real data, introducing bias and leading to incorrect conclusions about the estimated effects. These challenges prompt the use case for machine learning (ML) approaches that remove the requirement of manual specification and provide estimation of heterogeneous effects in a data-driven manner.

In the past decade, many ML methods have been proposed to address this need. Nonparametric tree-based methods, in both frequentist and bayesian frameworks, are some of the most developed and widely used approaches (Su et al., 2012; Athey & Imbens, 2016; Powers et al., 2018; Wager & Athey, 2018; Athey et al., 2019; H. A. Chipman et al., 2010; J. L. Hill, 2011; Hahn et al., 2020). Other methods traditionally used for prediction such as LASSO (Belloni et al., 2014; Imai & Ratkovic, 2013; Zhao et al., 2022) and neural networks (Shalit et al., 2017; Syrgkanis et al., 2019) have also been adapted for heterogeneous treatment effect estimation. Metalearners (Kennedy, 2023; Künzel et al., 2019; Nie & Wager, 2021) provide model-agnostic frameworks whereby any predictive algorithms can be combined in an ensemble to estimate heterogeneous effects. These approaches make use of a variety of estimation, inference, and analytical techniques and there is a growing body of literature comparing their performance and utility (Acharki et al., 2023; Caron et al., 2022; Dorie et al., 2019; A. Hu, 2023; Jacob, 2021; Liu, 2022; McConnell & Lindner, 2019; Wendling et al., 2018). However, this is a rapidly evolving field of study and there is a continuous need for interpretation of these methods, as well as guidance on applying them to real data. Furthermore, there is minimal guidance on how to use these methods for EMM analyses, particularly for public health applications. With few exceptions, these methods do not directly identify effect modifiers and estimate corresponding subgroup effects, which creates a disconnect for researchers interested in using these methods to circumvent the limitations of traditional methods. While a limited number of epidemiological studies used such approaches in the past few years, we are not aware of an up-to-date summary of some of the most commonly used methods as well as a guide about their implementation for EMM analyses using an illustrative case study.

In this paper, we summarize and provide the intuition behind modern ML approaches for EMM analyses in high-dimensional settings. These include bayesian additive regression trees, generalized random forests, and bayesian causal forests. While not an exhaustive list, these methods are the most commonly used ML methods in epidemiological studies at the time of this review. We discuss how these methods can be employed for EMM analyses, using supplemental techniques and tools to identify potential effect modifiers and estimate corresponding subgroup effects. While no data-driven method or technique can exhaustively identify effect modifiers and domain expertise is still necessary, these methods can serve an important role in the discovery of vulnerable subgroups when prior knowledge is not available. We discuss their implementation in R (R Core Team, 2023) with annotated syntax to serve as a reference for public health researchers interested in using these methods for their own EMM analyses. Lastly, we demonstrate the application of these methods by assessing the heterogeneous effects of drought on stunting among children from the Demographic and Health Survey (DHS) data as a case study.

In section 2, we provide the summaries of the ML methods. We organize these summaries into two categories: model-based and forest-based approaches. Section 3 discusses the implementation of the methods with the DHS data and provides several motivating examples. We conclude with a discussion in section 4.

## 2 EMM ML method overview

We first briefly introduce the terminology that will be used throughout this overview. The effect of an exposure A on a given outcome Y across a population is measured by the average treatment effect (ATE). To measure heterogeneous effects, the ATE is estimated within population subgroups. This quantity is called the conditional average treatment effect (CATE), as the ATE is conditional on covariates L that constitute relevant subgroups. If the CATE differs from the ATE, the corresponding subgroup is said to be heterogeneous with respect to the population.

Throughout this review, we assume the standard identification assumptions of conditional exchangeability, positivity, and consistency to make causal claims about the observed effects (Hernán, 2012). We provide more technical descriptions of the discussed estimands and assumptions in the appendix.

### 2.1 Model-based approaches

#### 2.1.1 Bayesian additive regression trees

**Fig. 1:**
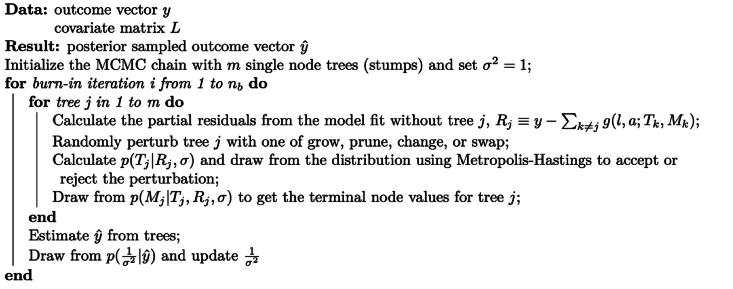
Bayesian Additive Regression Trees algorithm pseudocode.

Bayesian additive regression trees (BART) (H. A. Chipman et al., 2010) is the oldest method we consider. Like the traditional classification and regression tree (CART) algorithm (Breiman et al., 2017), BART is a nonparametric tree-based method that recursively partitions data to estimate the expected outcome conditional on partitioned covariates. However, BART is specified as an additive sum-of-trees model within a Bayesian framework. The additive model specification estimates linear relationships more accurately than individual tree models and attenuates interactions that individual tree models tend to overemphasize (J. L. Hill, 2011). Analogous to “boosting” in which multiple weak learners contribute to one strong learner, each tree is limited to small contributions to the overall BART model. This is accomplished through a regularization prior that limits the influence of individual trees. The model is fit using a Bayesian backfitting Markov Chain Monte Carlo (MCMC) algorithm summarized by Fig. 1.

**Fig. 2:**
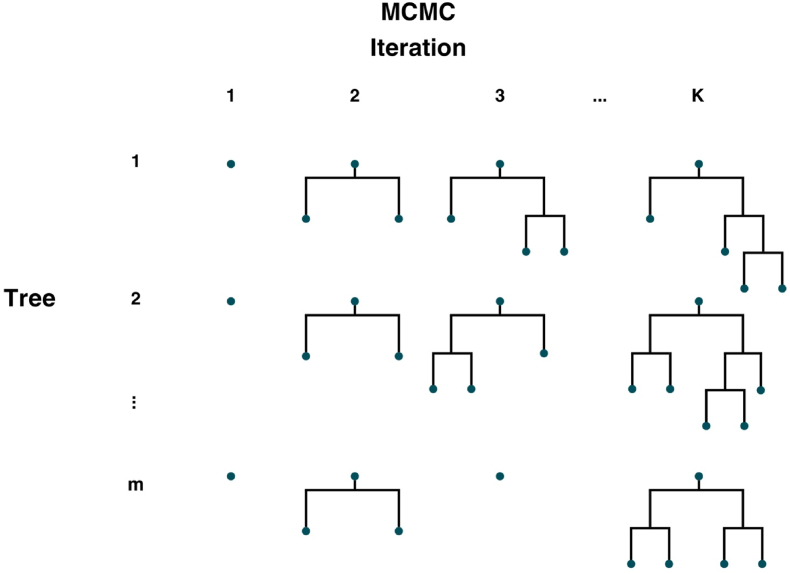
Illustration of the BART Bayesian backfitting MCMC algorithm, inspired by Hastie and Tibshirani (Stanford Online, 2022).

In each MCMC iteration, trees are randomly perturbed by one of four actions. The “grow” action assigns a covariate split to a terminal node at random, while the “prune” action removes the children of a random parent node. The “change” and “swap” actions alter the prediction of terminal nodes by either randomly replacing the splitting rule of an internal node or swapping the splitting rules of a random parent-child internal node pair. Rather than fitting new trees to partial residuals, the algorithm uses these perturbations and the defined priors to accept or reject the changes to each tree via the Metropolis-Hastings procedure. Fig. 2 illustrates this algorithm for m trees and K MCMC iterations.

As a standalone algorithm, BART only estimates the conditional expected outcome. However, Hill introduced BART as an effective tool for heterogeneous treatment effect estimation where one BART model is used to estimate potential outcomes and generate CATE estimates (J. L. Hill, 2011). This approach is referred to as the S-learner due to the use of a single model. In more recent years, other approaches such as the T-, X-, DR- and R-learners have been developed (Kennedy, 2023; Künzel et al., 2019; Nie & Wager, 2021). These approaches are known as metalearners and each performs optimally in different settings. We discuss the details of implementing BART with an S-learner in section 3.2.1 and provide a general discussion on the choice of metalearner in the appendix for interested readers.

Overall, the key property of BART is its applicability as an effective “off the shelf” method. This is due to the default regularization prior specification and its anti-overfitting nature. Parameter tuning and cross-validation are often not necessary and consequently, the method is computationally less taxing and simple to implement. Moreover, BART provides inference in the form of credible posterior intervals, which can be a more intuitive uncertainty metric than standard frequentist confidence intervals.

### 2.2 Forest-based approaches

#### 2.2.1 Generalized random forests

Generalized random forests (GRF) was proposed by Athey et al. (Athey et al., 2019) as the most recent development in a series of nonparametric tree-based methods for EMM (Athey & Imbens, 2016; Wager & Athey, 2018). The motivation behind these methods is twofold: data-driven estimation of heterogeneity and valid inference with confidence intervals for high-dimensional effect modification. GRF serves an abstraction to prior iterations and other common forest methods.

At a high level, GRF is a nonparametric tree-based method that recursively partitions data on covariate splits that maximize heterogeneity between nodes. The primary output of the method is the estimated CATE for each unit in the sample. GRF implements an “honest estimation” technique where the data used to train a forest is split into a set for tree growth and a set for CATE estimation. This reduces potential overfitting and decreases bias (Athey & Imbens, 2016), and contrasts with conventional machine learning training-test splits that are used to evaluate model performance.

GRF closely follows the Causal Forest (CF) algorithm (Wager & Athey, 2018), but additionally implements ‘orthogonalization’ via Robinson's transformation to better handle instances of strong confounding (Robinson, 1988). In such setting, conditional exchangeability can only be achieved for a CF by creating partitions on confounders. However, this can bias the CATE estimation if the covariates being split do not contribute to heterogeneity. Orthogonalization handles this problem by first modeling the propensity score and marginal outcome functions to account for the effects of confounding. The residual treatment and outcomes are then computed and used to train a CF, allowing the partitioning to focus on covariates that contribute to effect modification.

**Fig. 3:**
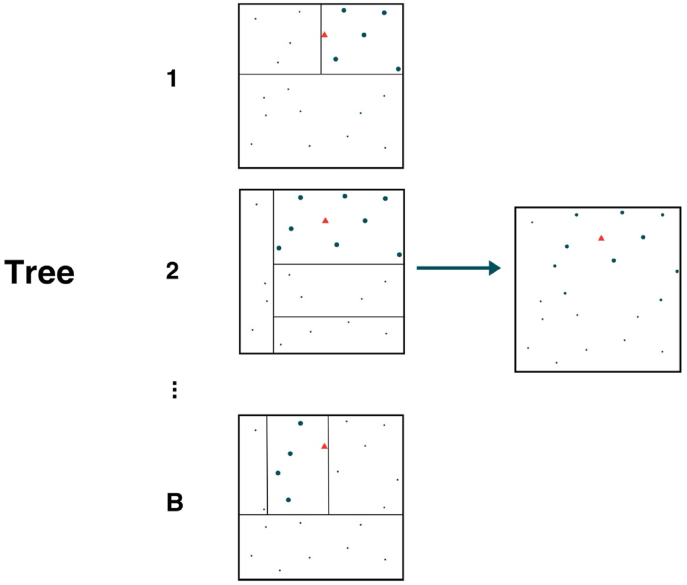
Visualization of the Generalized Random Forest weighting procedure, inspired by Fig. 1 from Athey et al. (Athey et al., 2019).

**Fig. 4:**
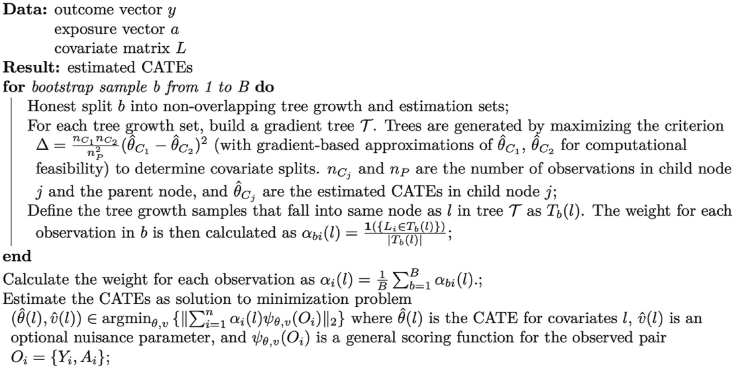
Generalized Random Forests algorithm pseudocode.

Additionally, unlike standard forest methods that average predictions across trees (i.e. “bagging”), GRF uses an adaptive kernel method that performs locally weighted optimization to estimate CATEs. Each unit is assigned a weight that represents the frequency with which the i th sample falls in the same terminal node as l across all trees in the forest. These weights depict neighborhoods with similar observations to l and contribute to more stable estimates than averaging (Athey et al., 2019). Fig. 3 illustrates the weighting process. Each square in the left column depicts a tree in a forest with internal boxes representing terminal node partitions. The given l is shown by a red triangle and the square in the right column shows the final observations with node sizes relative to their weights. The algorithm steps are summarized in Fig. 4.

#### 2.2.2 Bayesian causal forests

Bayesian causal forests (BCF) aims to improve upon BART in a similar sum-of-trees model (Hahn et al., 2020). The authors identified two issues with BART: “regularization-induced confounding” and high CATE estimate variability in the presence of homogeneity or moderate heterogeneity. Regularization-induced confounding occurs when confounders are regularized out of the model outcome surface due to their lack of predictive power (Hahn et al., 2018). This is an issue when the confounders serve the role of reducing bias in the exposure-outcome relationship. Hahn et al. propose including an estimate of the propensity score as a covariate to address this issue. High CATE estimate variability occurs when the heterogeneity signal is low, and the authors propose reparameterizing the outcome surface into two independent BART ensembles as a solution: one prognostic component that models the relationship between the covariates and outcome, and another component that models the CATE.

This reparameterization allows for different covariate sets for the two BART components, which is beneficial when it is known that the set of confounders and effect modifiers are not equivalent. The number of trees can also differ between the components, enabling users to customize the complexity of the effects (McJames et al., 2023). Lastly, the components have independent BART priors that can provide different regularization. The default prior for the CATE component provides stronger regularization than the prior for the prognostic component, favoring homogeneity unless there is strong evidence to the contrary.

The Bayesian backfitting MCMC algorithm for BCF is identical to that of BART, with slightly modified priors and two BART models run within each MCMC iteration. Overall, BCF provides adjustments for identified problems with standard BART and seeks to improve the accuracy of CATE estimation.

## 3 Implementation of the selected EMM ML methods

In this section, we review the currently available tools to implement these methods in R version 4.3.2 (R Core Team, 2023) and apply them to a case study related to the effect of drought on stunting using the Demographic and Health Survey (DHS) data. We also discuss common techniques to identify potential effect modifiers and estimate corresponding subgroup effects. We then compare the results from these methods to those of traditional methods. All tunable parameters for each method were left at their defaults and the code for these implementations can be found on GitHub (https://github.com/benmarhnia-lab/EMM-ML) and in the appendix.

### 3.1 Data

We use the DHS data set to demonstrate the application of these methods. These data focus on women in reproductive age (ages 15 to 49) and their children under 5 years of age in low- and middle-income countries (LMIC) and cover a wide range of health-related issues, including fertility, mortality, diseases, nutrition, and health-seeking behavior. A more detailed description of the data is provided in the appendix.

The outcome in our analysis is stunted child growth and the exposure is drought. The covariates are child sex, age, birth size, breastfed status, the mother's education level, single status, occupation, and family media consumption, rural residence, and wealth level. We choose this set of covariates for the purpose of demonstration and simplicity. However, the methods and corresponding interpretation are generalizable to larger covariate sets and higher dimensions of EMM. Table 1 lists the variables used in this analysis and their prevalence in the data. All variables are coded as binary indicators and missingness has been removed such that all observations are complete.

**Table 1:** Descriptive statistics.

	Stunted Child Growth		
Variable	Overall, N = 345,499^a^	Not Stunted, N = 212190 (61%)^*1*^	Stunted, N = 133309 (39%)^a^
Exposure			
Drought	50,310 (15%)	30,034 (14%)	20,276 (15%)
Covariates (Effect Modifiers/Confounders)			
Child Sex - Male	174,324 (50%)	103,099 (49%)	71,225 (53%)
Child Age - Under 2	127,378 (37%)	84,656 (40%)	42,722 (32%)
Child Birth Size - Small	62,218 (18%)	34,449 (16%)	27,769 (21%)
Child Breastfed - Never	7463 (2.2%)	4558 (2.1%)	2905 (2.2%)
Mother's Education - None	174,528 (51%)	99,828 (47%)	74,700 (56%)
Mass Media Consumption - Yes	156,872 (45%)	105,636 (50%)	51,236 (38%)
Single Mother - Yes	27,128 (7.9%)	16,348 (7.7%)	10,780 (8.1%)
Agricultural Occupation - Yes	161,724 (47%)	87,741 (41%)	73,983 (55%)
Residence - Rural	249,258 (72%)	142,971 (67%)	106,287 (80%)
Wealth - Poor	158,259 (46%)	83,998 (40%)	74,261 (56%)

All variables recorded as binary.

a n (%).

### 3.2 ML methods

#### 3.2.1 Bayesian additive regression trees

Several R packages exist to implement BART. The most prominent include *dbarts* (Dorie et al., 2014) and its corresponding causal inference derivative *bartCause* (J. L. Hill, 2011), and *BART* (Sparapani et al., 2021). These packages are modern iterations of *BayesTree* (H. Chipman & McCulloch, 2006) and *bartMachine* (Kapelner & Bleich, 2016). We choose *BART* for our application, but any of these packages may be used to implement BART. The function *wbart* generates the model for continuous outcomes while *lbart* and *pbart* are used for dichotomous outcomes (logistic and probit links, respectively).

We implement BART using an S-learner to follow the original framework with which BART was proposed for estimating CATEs (J. L. Hill, 2011) and for the purpose of demonstration. We also choose an S-learner for our demonstration to conform with the majority of examples in the literature. 2 counterfactual data sets are created where the exposure is present or absent for all observations. The BART model instance *wbart* can then be trained on the full sample with the counterfactual data sets used to estimate the potential outcomes. The required inputs to the function are the matrix of covariates (including the exposure) and outcome vector to train the model, and the matrix of counterfactual covariates. The vector of CATE estimates can be obtained by averaging the estimated potential outcomes across MCMC iterations and taking the difference between the exposed and unexposed sets. For binary or other outcomes in which the link function is not the identity, CATEs may be transformed back to the additive scale to estimate absolute risk (for a binary outcome, we use the inverse logit function expit(x)=1/(1+exp(−x))). Tuneable parameters of note are the number of burn-in MCMC iterations as the threshold for posterior convergence and the number of MCMC iterations to save after burn-in.

To identify potential effect modifiers and estimate corresponding subgroup effects, a technique known as “fit-the-fit” is often performed in which the estimated CATEs are modeled as a function of the covariates in a CART model. This allows for efficient identification of covariates that contribute most to the variability of the CATE estimates and provides effect estimates of the subgroups defined by the covariate splits. This technique is often implemented with a maximum node depth of 3 so that the corresponding subgroups are meaningful (J. Hill & Su, 2013).

**Fig. 5:**
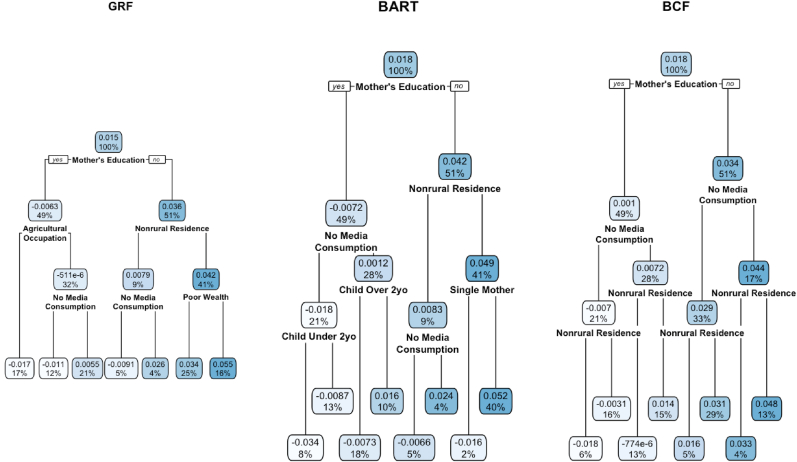
Fit-the-fit CARTs (classification and regression trees) of GRF, BART, and BCF. The value at the top of each box gives the estimated treatment effect for the subgroup defined by the splitting rules. The value at the bottom of each box represents the proportion of units in the subgroup.

Fig. 5 shows the fit-the-fit CART of the estimated CATEs from BART with a set maximum node depth of 3. The CATEs are first split on maternal education with an estimated ATE of 1.8%. 49% of the observations have some maternal education with an exposure effect of −0.72% while 51% of the observations have no maternal education and an effect of 4.2%. Residence status is also represented in a level 1 node split with subgroup effects of 0.83% and 4.9%, corresponding to the two-way interaction of no maternal education and non-rural or rural status.

Example applications of BART for EMM analysis with real-world data can be found in Blette et al., Hu et al., and Kraamwinkel et al. (Blette et al., 2023; L. Hu et al., 2021; Kraamwinkel et al., 2019). Blette et al. conducted a post hoc heterogeneity analysis of the COVID STEROID 2 trial, which compared 6 mg/d to 12 mg/d of dexamethasone for patients with severe or critical COVID-19. In the original trial, subgroup analyses were conducted with prespecified covariates, and no subgroup effects were found to be statistically significant at the P<.01 threshold adjusted for multiple comparisons. Using BART in an S-learner framework and fit-the-fit to identify potential effect modifiers, the authors found that individuals who needed higher level respiratory support had a greater benefit from the higher dexamethasone dose compared to the study population. Moreover, those treated with IL-6 inhibitors had less benefit from the higher dose. Hu et al. also conducted a post hoc analysis of data from a lung cancer trial, looking at the heterogeneous effects of low-dose computed tomography compared to chest radiography on survival rates. BART with an S-learner and fit-the-fit were used to reveal potential racial disparities in the overall mortality benefit. Lastly, Kraamwinkel et al. employed BART with an S-learner to analyze the effect of maternal education on severe child undernutrition using the DHS data. Despite the literature suggesting armed conflict as a potential effect modifier, the authors did not find it produced significant effect modification. These examples highlight the utility of BART for discovering potential effect modifiers and vulnerable subgroups.

#### 3.2.2 Generalized random forests

GRF is implemented with the *causal_forest* function in the R package *grf* (Tibshirani et al., 2017). The required inputs for the function are the matrix of covariates, the outcome vector, and the exposure vector. By default, *causal_forest* implements orthogonalization described in the prior section. Notable parameters include the number of trees grown in the forest, minimum node size, and honest splitting ratio, which can be tuned via the *tune_parameters* argument. After the forest has been generated, the *predict* function is used to obtain the output vector of CATEs expressed as risk differences and *average_treatment_effect* can be used to estimate the ATE.

To assess overall heterogeneity, a “best linear predictor” (BLP) analysis may be conducted with the function *test_calibration*. This serves as an omnibus calibration test of the quality of the CATE estimates and presence of heterogeneity within the data (Athey & Wager, 2019; Chernozhukov et al., 2018). Results are presented as regression coefficients for *mean* and *differential forest predictions*. The coefficient for the *mean forest prediction* represents the quality of the ATE estimate while the coefficient of the *differential forest prediction* represents the quality of the CATE estimates. When the coefficient is close to 1 in magnitude, the ATE or CATEs are well calibrated, respectfully. Moreover, if the coefficients are positive and significant, then there is evidence to reject the null hypothesis of no treatment effect or heterogeneity in the data. An informal calibration test may also be conducted by grouping the CATE estimates into quantiles and visually observing the trend in the average CATE values across quantiles (Shiba & Inoue, 2024). If the forest is well-calibrated, the average CATEs should monotonically increase across quantiles.

In addition to the fit-the-fit procedure, variable importance can be used to readily identify potential effect modifiers. This metric, provided by the function *variable_importance,* represents the weighted sum of the number of times each covariate was used in a tree node split. A large variable importance value indicates large influence in the CATE estimation, suggesting the corresponding covariate may be a strong modifier of the treatment effect. However, it is crucial to note that variable importance does not equate to theoretical relevance as an effect modifier. While there are no strict guidelines that dictate how covariates should be identified, it is common to view covariates that have a metric value above a given threshold or the mean value as potential effect modifiers (Athey & Wager, 2019). With this information, a second stage regression analysis can be performed with the function *best_linear_projection* that regresses the CATE estimates on prespecified covariates. The resulting model coefficients are doubly robust effect estimates for subgroups defined by the provided covariates.

It is also common to conduct descriptive analyses of the CATEs for predefined subgroups to investigate potential heterogeneity. For example, one may plot the CATE distribution across subgroups to visualize differences. Unlike traditional analyses that require discrete effect modifier definitions, the CATEs can be plotted across continuous covariates to reveal potential nonlinearities (Shiba & Inoue, 2024). Lastly, one can rank the CATEs by quantile and compare the mean CATE values across covariates. This is commonly performed with quintiles, the median (i.e. above or below the 50th percentile), or the top 10% against the bottom 10%. However, these techniques require manual specification of potential effect modifiers and are subject to the problem of multiple comparisons.

Table 2 shows the results from applying the BLP test, second stage regression analyses, and lists the variable importance for each covariate. The mean forest prediction coefficient is close to 1 in magnitude and statistically significant, indicating that there is an overall effect of drought on stunted child growth measured by the ATE and the GRF captures this effect well. The coefficient for the differential forest prediction is statistically significant but not close to 1 in magnitude. This implies that heterogeneity exists in the data, but it was not accurately captured by the GRF CATEs. For demonstration, we perform second stage regression on the subgroups of the covariates with over 10% variable importance. We see that maternal education and residence status are the most important covariates in the forest. The second stage regression finds that the exposure effects for those whose mothers have no education and those who live in a rural area are 4.2% (95% CI: [3.3%, 5.1%]) and 1.9% (95% CI: [0.9%, 2.9%]), respectively. The GRF fit-the-fit CART is shown in Fig. 5. Maternal education is again the first covariate split used and the subgroup effects are similar to those from BART. Rural residence features as level 1 splitting variable and the subgroup effects agree with the BART results.

**Table 2:** Generalized Random Forests results.

Estimand	Estimate (p-value or 95% CI)
Best Linear Predictor Calibration	
Mean forest prediction	0.988 (<0.001)
Differential forest prediction	0.385 (<0.001)
Second Stage Regression	
Mother's Education - None	0.042 (0.033, 0.051)
Residence - Rural	0.019 (0.009, 0.029)
Variable Importance	
Mother's Education	61.9%
Residence	11.1%
Mass Media Consumption	6%
Single Mother	4.5%
Agricultural Occupation	3.9%
Child Age	3.6%
Child Birth Size	3.6%
Wealth	3.2%
Child Sex	2.2%
Child Breastfed	0%

Examples of GRF can be seen in papers such as Shiba et al., Naito et al., and Matsuyama et al. (Matsuyama et al., 2024; Naito et al., 2024; Shiba et al., 2021, 2023) Shiba et al. used GRF to examine the heterogeneous effects of disaster-related home loss on cognitive disability and functional limitations in older adults. To identify potential effect modifiers, they compared the top decile of the estimated CATE distribution to the bottom decile across available covariates. Additionally, they compared the CATE distributions across the covariates with the 3 largest variable importance metrics for the functional limitation outcome. They found that the most vulnerable individuals were older, not married, living alone, less educated, and had more health problems. Surprisingly, they also found that vulnerable individuals were likely to have higher income when paired with less education and more health problems. While the literature suggests a protective effect of high income, the authors were able to uncover complex effect modification in the opposing direction using these approaches. Naito et al. demonstrated heterogeneous associations of environmental risk factors and cardiometabolic diseases across age, sex, and polygenic risk score by comparing the top decile to the bottom decile of the CATEs estimated by GRF. Lastly, Matsuyama et al. analyzed the heterogenous effects of tooth loss on functional capacity and found larger effects for individuals who were older men, did not have a partner, had poor health, and were of lower socioeconomic status.

#### 3.2.3 Bayesian causal forests

BCF is implemented with the package and identically named function *bcf* (Hahn et al., 2020). The required inputs are the matrix of covariates, outcome vector, exposure vector, and estimated propensity score. We choose to estimate the propensity score with a logistic regression model where the exposure is modeled as a function of the additive effects of the covariates for simplicity. Like BART, the burn-in MCMC iteration threshold and the number of saved MCMC iterations after burn-in are tuneable parameters, and the CATE estimates expressed as risk differences are obtained by averaging the posterior samples. The primary tool to identify potential effect modifiers and estimate subgroup effects using BCF is the fit-the-fit CART approach. Currently, we are not aware of other example applications of BCF for EMM analysis using real-world data.

Fig. 5 shows the BCF CART. The covariate used in the first split rule is maternal education and the subgroup effects are similar to those from the GRF and BART CARTs. Unlike the GRF and BART CARTs, rural residence is not used as a level 1 splitting variable, but it is used as a level 2 splitting variable for all nodes in the tree.

#### 3.2.4 Comparison of approaches to identify effect heterogeneity

The most common technique to identify potential effect modifiers and estimate corresponding subgroup effects using these methods is the fit-the-fit CART. While crude, this technique does not require prior knowledge of effect modifiers or manual specification. Variable importance metrics can also be used to identify potential effect modifiers and second stage regression or CATE quantile comparisons allows for effect estimation of specified subgroups.

**Fig. 6:**
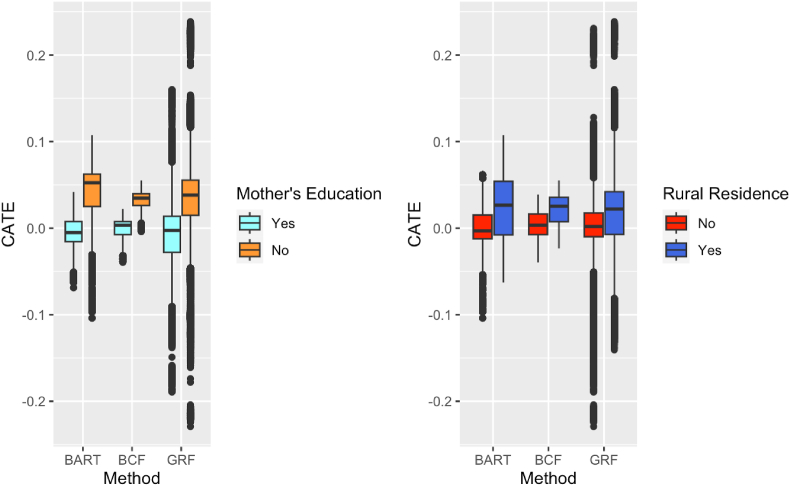
CATE comparison for GRF/BART/BCF across levels of mother's education and rural residence.

Fig. 6 shows each method's CATE distribution for different subgroups of potential effect modifiers. For all three methods, there is a visible increased risk for units with no maternal education compared to those who had education, and for those whose residence status is rural compared to non-rural.

### 3.3 Comparison with traditional methods

As discussed in section 1, traditional methods for EMM analysis involve either stratified analyses or a multivariable model with interaction terms. We conduct a stratified analysis of logistic regression models adjusted for all covariates with subgroup effects represented by odds ratios. To compare results to those from the ML methods, we use the subgroups defined by the two levels of maternal education. We also conduct Cochran's Q test to determine if there is evidence to reject the null hypothesis of no heterogeneity.

Table 3 gives the estimands and corresponding 95% confidence intervals for the traditional analysis methods and results from Cochran's chi-squared test for heterogeneity. The first two rows give the unadjusted risk difference and risk ratio estimates in the full data, no maternal education subgroup, and some maternal education subgroup. We find a significant increased risk of stunted child growth due to drought in the full sample (RD: 2% [1.5%, 2.5%]; RR: 1.05 [1.04, 1.07]). The risk is larger in the no maternal education subgroup (RD: 3.4% [2.8%, 4.1%]; RR: 1.08 [1.07, 1.1]), and there is no strong evidence of an increased risk in the some maternal subgroup (RD: −0.7% [−1.4%, 0%]; RR: 0.98 [0.96, 1]). The third row gives the ATE in the full sample and effects from the maternal education subgroups. These estimates also suggest a significant increased risk of stunted child growth due to drought in the full sample (OR: 1.07 [1.05, 1.09]) and a larger risk in the no maternal education subgroup (OR: 1.15 [1.12, 1.18]). Again, we find no strong evidence of an effect of drought on stunted child growth in the some maternal education subgroup (OR: 0.97 [0.94, 1]). Cochran's Q test suggests there is evidence to reject the null hypothesis of no EMM between the maternal education subgroups (*P* < 0.001). These findings agree with those found by the ML methods and strengthen the case for maternal education as an effect modifier.

**Table 3:** Traditional methods results.

Estimand	Group		
	Full Sample	Mother's Education - None	Mother's Education - Some
Risk Difference (95% CI)	0.02 (0.015, 0.025)	0.034 (0.028, 0.041)	−0.007 (−0.014, 0)
Risk Ratio (95% CI)	1.053 (1.04, 1.065)	1.081 (1.066, 1.096)	0.979 (0.96, 0.999)
(C)ATE (95% CI)^a^^,^^b^	1.066 (1.045, 1.087)	1.151 (1.121, 1.182)	0.97 (0.941, 1)
Cochran's Q test statistic (p-value)		65.492 (<0.001)	65.492 (<0.001)

a Expressed as odds ratio.

b Adjusted for covariates.

## 4 Discussion

In this overview, we summarized three recently developed ML methods for EMM analysis that have been used in various quantitative social sciences disciplines but to a lesser extent in epidemiology data. These nonparametric, data-driven methods allow for flexible modeling of nonlinear outcome surfaces and high-dimensional interactions that are impractical to test by hand. However, the methods do not identify effect modifiers and estimate corresponding subgroup effects. We discussed their current implementation in R, the common techniques used to identify potential effect modifiers and estimate corresponding subgroup effects, and applied them using a case study focusing on the effect of drought on stunting among children in multiple Sub-Saharan countries. With these techniques, the ML methods identified maternal education as a potential effect modifier, which may have been overlooked or obscured by a multiple comparisons adjustment had only traditional methods been used.

“Fit-the-fit” CART and variable importance are tools that are often used to efficiently identify potential effect modifiers. However, these techniques do not guarantee that identified covariates are true effect modifiers (variables that would modulate the amplitude of the effect estimate). Covariates may have large influence in fit-the-fit CART models or large variable importance due to high correlation with other effect modifiers (Jawadekar et al., 2023). Variable importance should not be interpreted as the proportional influence of heterogeneity or the likelihood of a covariate being a true effect modifier. In addition, the assessment of potential effect modifiers is driven by data availability, and it is worth mentioning that in some settings, a given effect modifier may be correlated with other unmeasured effect modifiers. This reinforces the importance of distinguishing the concepts of EMM and causal interaction as no manipulation is required for EMM analyses. These tools are helpful for identifying covariates for which variability in the effect estimates is high with no prior knowledge of true effect modifiers. Second stage regression and descriptive analyses such as plotting CATE distributions across covariate groups are also used to assess EMM but require manual specification of potential effect modifiers.

It is important to emphasize that no algorithm can automatically select what constitutes a true effect modifier. The methods and techniques discussed in this overview only provide data-driven results insofar as modeling the CATE and highlighting covariates that are associated with CATE estimates. Selecting effect modifiers must be based on pre-existing knowledge regarding a specific exposure-outcome relationship. While the concepts of confounding and effect modification are fundamentally different, it is known that confounders (that are minimally associated with the outcome of interest, without being a collider variable) constitute effect modifiers on at least one scale (additive or multiplicative) (Rothman et al., 2021). Therefore, we suggest it is reasonable to consider all confounders (and their multiple combinations and functional forms) as potential effect modifiers when exploring high-dimensional EMM analyses. Furthermore, methods such as those discussed in this paper are often designed to estimate effects on an additive scale and not a multiplicative scale. As previously mentioned, heterogeneity of effects is scale-dependent and it is recommended to report effects on both scales (Kent et al., 2020; VanderWeele & Knol, 2014). Therefore, researchers may consider methods or frameworks such as the S- or T-learner that estimate potential outcomes to construct effect estimates on a multiplicative scale.

It is also important to differentiate between estimation strategies and estimators. Metalearners such as those discussed in the previous sections are CATE estimators while methods like BART are predictive algorithms that can be specified as base learners. Moreover, there has been a recent emphasis on classifying methods for CATE estimation using the metalearner typology. For example, GRF may be seen as a special case of the R-learner that uses regression forests as base learners. Nonetheless, there are several studies that demonstrate how estimates can vary widely across estimators and estimation strategies (Bouvier et al., 2024; Jacob, 2021; W. Zhang et al., 2022), indicating that varying specifications of both are worthy of comparison.

We have not discussed other methods that may be used to identify heterogeneous subgroups and estimate CATEs due to their novelty and lack of use in empirical settings. However, we would like to mention some recently developed alternative approaches. In particular, the causal rule ensemble (CRE) (Bargagli-Stoffi et al., 2024), multilevel analysis of individual heterogeneity and discriminatory accuracy (MAIHDA) (Rodriguez-Lopez et al., 2023), and the iterative Causal Forest (iCF) (T. Wang et al., 2024) are methods that can automate effect modifier identification. The CRE accomplishes this through a procedure of estimating CATEs, generating heterogeneous subgroups from the CATE estimates, and selecting the most important heterogeneous subgroups through penalized regression. Several ML methods are used in this ensemble procedure such as random forests and LASSO, and any of GRF, BART, or BCF may be used during the CATE estimation step. MAIHDA implements a mixed-effects regression model where subgroups are treated as random-intercepts. Heterogeneous subgroups can be identified by the magnitude of the estimated random effects and CATEs are estimated as linear combinations of fixed and random effects. Lastly, iCF utilizes GRF to build and select the best trees from forests at different low-dimensional depths, resulting in heterogeneous subgroups defined by the splitting rules. These methods are not commonly used for EMM analyses yet but are promising tools for automating heterogeneous subgroup identification.

There are several limitations of this overview. We chose to implement these methods in a simple setting with a small set of covariates and default specifications that have been used in applied studies. While the methods theoretically extend to larger data sets, they have not been used extensively with real-world, high-dimensional data to the best of our knowledge. However, examples such as Waldmann (who used BART to identify single-nucleotide polymorphisms that contributed most to genome-wide prediction) show the potential of these methods in high-dimensional settings (Waldmann, 2016). Moreover, we did not evaluate the performance of these methods or provide in-depth theoretical explanations of the different estimation strategies. While we aim to provide a practical, straightforward tutorial of these methods, we acknowledge these discussions are beyond the scope of this paper and refer to others for more detail (Athey et al., 2019; Chernozhukov et al., 2024; H. A. Chipman et al., 2010; Hahn et al., 2020). We also chose to demonstrate the application of these methods using observational data. However, post-hoc heterogeneity analyses of clinical trial data are often underpowered because the data are collected to power the main treatment effect (Brookes et al., 2004). Extending these methods outside of the observational setting to clinical trial data with techniques such as data fusion and integration remains a methodological challenge (L. Zhang et al., 2018). Ultimately, all the considered methods are useful tools for exploring heterogeneity within real world data. The data-driven nature of these methods distinguishes them as helpful tools for initial exploration of effect modification, while traditional methods better serve as tools for confirmatory analyses.

Machine learning for effect measure modification is a burgeoning and promising field of study. As such, there is a constant need for simple interpretation of newly developed tools to increase their accessibility for applied researchers. This overview provides this interpretation and guides readers on implementing these tools and other supplemental analysis techniques in their own research. We hope that it serves as a useful reference for researchers in public health and adjacent disciplines.

## CRediT authorship contribution statement

**Michael Cheung:** Writing – review & editing, Writing – original draft, Visualization, Software, Formal analysis, Conceptualization, Methodology. **Anna Dimitrova:** Writing – original draft, Data curation. **Tarik Benmarhnia:** Writing – review & editing, Supervision, Methodology, Funding acquisition, Conceptualization, Writing – original draft.

## Reproducibility process

The data used in this manuscript come from the Demographic and Health Survey data set. These data are publicly available, and we provide a description of how we cleaned and formatted the data for our analyses. We provide annotated code in R to replicate the results of our analyses in the appendix.

## Ethical statement

The data used in this study were from the Demographic and Health Surveys program, which complies with the U.S. Department of Health and Human Services regulations for the protection of human subjects.

## Source of funding

The National Institute on Aging (RF1AG080948).

## Declaration of competing interest

The authors declare that they have no known competing financial interests or personal relationships that could have appeared to influence the work reported in this paper.

## Figures and Tables

**Figure:**
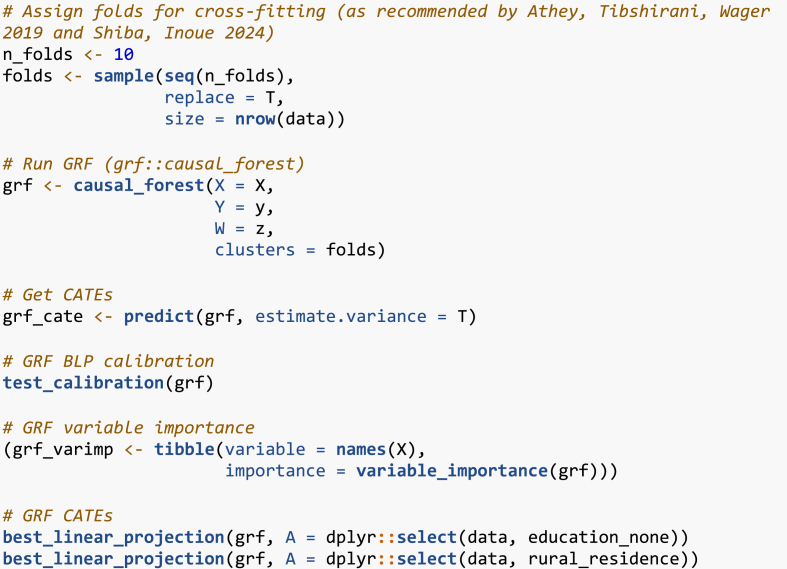


**Figure:**
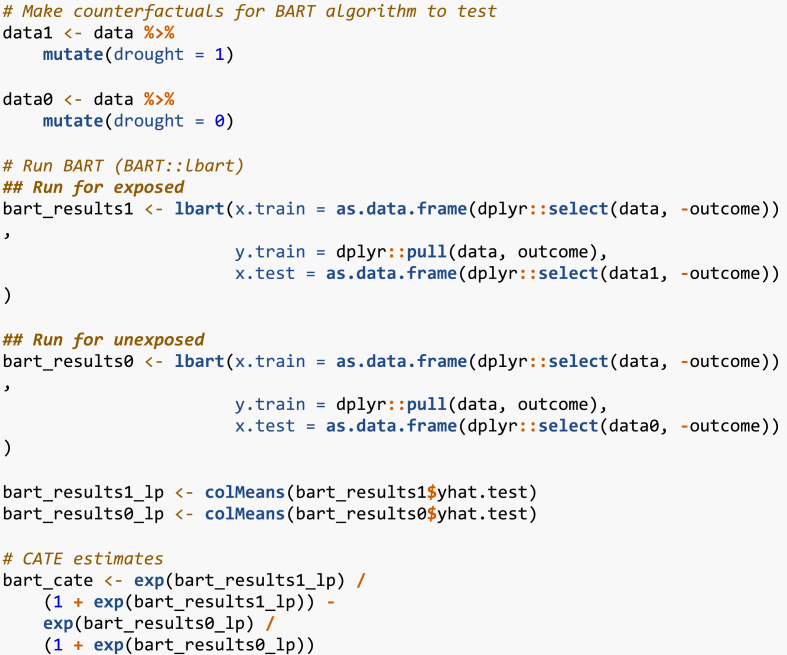


**Figure:**
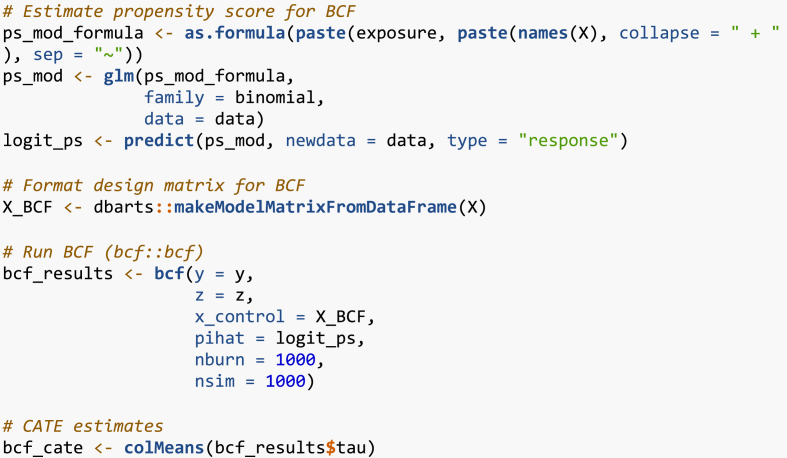


## Data Availability

I have shared the link to the data at the Attach File step. Githubdata_droughts_malnutrition.csv (Reference data)

## Appendix

### A.1 Technical definitions and assumptions for valid causal inference

Under Neyman-Rubin's potential outcomes framework (Rubin, 1974), let $Y_{i}\left(A_{i}\right)$ be the potential outcome for the exposure $A_{i}=0,1$ and $L_{i}$ be the covariate values for unit i=1,...,n. The ATE is defined as $E\left[Y_{i}\left(1\right)-Y_{i}\left(0\right)\right]$ and the CATE is defined as $\tau \left(l\right)=E\left[Y_{i}\left(1\right)-Y_{i}\left(0\right)|L_{i}=l\right]$ for covariates $l\in R^{p}$.

We assume the standard identification assumptions of conditional exchangeability, positivity, and consistency to make claims about observed effects (Hernán, 2012).

Conditional exchangeability states that the potential outcomes for both levels of the exposure are independent of the observed exposure conditional on observed covariates. Formally, $Y_{i}\left(1\right),Y_{i}\left(0\right)⊥A_{i}|L_{i}\text{.}$ This assumption is also referred to as the assumption of no unmeasured confounding.

Positivity guarantees that the conditional probability of being exposed or unexposed is greater than zero for any set of observed covariates, i.e., $P\left(A_{i}=a|L_{i}=l\right)>0$.

Lastly, consistency states that the observed outcome is equal to the potential outcome that would have been observed if the exposure took the same value. For a two-level exposure, $Y_{i}=A_{i}Y_{i}\left(1\right)+\left(1-A_{i}\right)Y_{i}\left(0\right)$.

### A.2 BART and BCF model definitions

BART is modeled as

$$Y=f\left(a,l\right)+\epsilon =\sum_{j=1}^{m}g\left(a,l;T_{j},M_{j}\right)+\epsilon$$

where $\left(T_{j},M_{j}\right)$ is the binary tree $T_{j}$ with corresponding b terminal node parameters $M_{j}=\left\{\mu_{1},...,\mu_{b}\right\}$ for trees j=1,…,m, and $\epsilon ∼N\left(0,\sigma^{2}\right)$. $g\left(a,l;T_{j},M_{j}\right)$ is the parameter associated with the terminal node that contains the pair (a,l) for tree $\left(T_{j},M_{j}\right)$.

The BCF model is expressed as

$$Y=f\left(a,l\right)+\epsilon =\mu \left(l,\hat{\pi }\left(l\right)\right)+\tau \left(l\right)a+\epsilon$$

where μ is the BART component that explains the relationship between l and Y, and τ is the BART component that represents the CATE.

### A.3 Choice of metalearner

To estimate CATEs, a metalearner such as the S-, T-, X-, DR-, or R-learner may be used. The choice of metalearner is context dependent and no metalearner outperforms all other options in every setting (Chernozhukov et al., 2024). Briefly, the S-learner trains a single model and treats the exposure variable as another covariate, resulting in instances in which the exposure is regularized out of the outcome surface and ignored entirely. It is simple to implement and preferable when the true CATE function is frequently zero. However, this can result in estimated CATEs being biased towards zero when the true function is nonzero. The T-learner is more appropriate for estimating weak CATEs with complex outcome surfaces but does not perform as well when the surfaces are simple and similar between the exposed and unexposed groups. This is a consequence of imbalanced designs which are common in observational data. The X-learner handles imbalanced designs better than the S- and T-learners by weighting the exposed and unexposed outcome surfaces by the propensity score. If the CATE function is simpler than the exposed or unexposed outcome surfaces, the X-learner is preferable to other metalearners. Lastly, unlike the S-, T-, and X-learners, the DR- and R-learners are optimized to estimate the CATE function rather than outcome surfaces. This is beneficial when the true CATE function is complex, particularly more than the outcome surfaces. The DR-learner is doubly robust such that only one of the propensity or outcome model needs to be accurately learned to achieve accurate CATE estimates. However, the DR-learner can be unstable and produce large variances when the true propensities include extreme values. Conversely, the R-learner is more stable in the presence of extreme propensities but is more dependent on accurate propensity score modeling than the DR-learner. We refer to others, in particular Salditt et al., for a full tutorial on implementation of these metalearners (Salditt et al., 2024).

### A.7 Description of Data

The DHS surveys are repeated cross-sectional surveys that have been collected in over 90 low- and middle-income countries (LMIC) since the 1980s. These data focus on women in reproductive age (ages 15 to 49) and their children and cover a wide range of health-related issues, including fertility, mortality, diseases, nutrition, and health-seeking behavior. The surveys also include detailed socioeconomic information, such as household assets, urban or rural places of residence, and type of occupation, among other information. A two-stage cluster sampling process guarantees that the data collected is nationally representative. More recent survey rounds include global positioning system (GPS) information (latitude and longitude) for each primary sampling unit (PSU). A PSU is defined as a city block in an urban area and a village in a rural area. We were able to connect the survey data with high-resolution gridded climate data via the GPS information.

For our analysis, we restrict the data to surveys collected between 2000 and 2022 in sub-Saharan African countries. We focus on children under 5 years of age for whom detailed information was collected, including anthropometric measurements (weight and height), birth weight, and feeding practices. To reduce the risk of misreporting bias, we restrict the sample to children who live with their mothers and are usual residents at the place of interview. Our final sample consists of 345,499 children from 32 countries and 86 individual surveys.

Following standard practice, we construct a binary indicator for child stunting based on the children's height-for-age z-scores (HAZ). Children are classified as stunted if their HAZ score is more than 2 standard deviations below the World Health Organization's (WHO) growth standard median for children of the same age-group (World Health Organization, 2019). We dropped observations with implausible HAZ scores (below −6 and above 6). Low HAZ values reflect chronic undernutrition in children (stunted growth) (De Onis, 2017). It is a serious health concern since stunted growth has been associated with a range of negative outcomes such as diminished cognitive function, learning challenges and an increased risk of chronic health conditions (Arthur et al., 2015; Black et al., 2013; Poveda et al., 2021; Prendergast & Humphrey, 2014; Victora et al., 2008). Nearly a third of children in sub-Saharan African are stunted – some of the highest levels observed worldwide (Levels and Trends in Child Malnutrition Child Malnutrition, 2023).

In addition to children's anthropometric measurements, we retrieve information about the demographic and socioeconomic characteristics of the children and their households, including the mother's level of education (grouped into none or some education), the type of occupation of the household head (agricultural or non-agricultural), the location of the household (urban or rural), mother's consumption of mass media (including television, radio and newspaper), and household's relative wealth group (constructed based on standard DHS procedures) (Rutstein, 2015).

To assess the impact of climate shocks on the health status of LMIC children under 5, we merged the survey data with Standardized Precipitation Evapotranspiration Index (SPEI), a multi-scalar drought index that is commonly used to measure the intensity and spatial distribution of droughts. The SPEI index is computed using input monthly precipitation and potential evapotranspiration data from the Climatic Research Unit at the University of East Anglia (CRU TS4) (Harris et al., 2020). We focus on identifying droughts during key agricultural periods. In particular, the main crop growing period for each PSU location is identified using high-resolution gridded data on the geographical distribution of crop areas (Monfreda et al., 2008) and crop calendar information (Sacks et al., 2010). Following standard practice, droughts are defined as crop-growing period SPEI values below −1. We focus on exposure to agricultural droughts during the infancy period (the first 12 months of life) since this is a period when most growth faltering is shown to occur (Alderman & Headey, 2018).

There is strong evidence that droughts pose a serious threat to child health and nutrition in LMICs (Belesova et al., 2019; Cooper et al., 2019). However, the population groups most at risk have not been well studied. The extent to which climate shocks impact human health is likely to vary depending on the underlying level of population vulnerability. The degree to which agricultural droughts disrupt livelihoods and have an impact on human health depends on both individual and community-level factors, including dependence on agricultural produce, the capacity to diversify income, and access to information, to mention a few. Identifying population groups that are particularly vulnerable to climactic shocks is important for designing targeted interventions. Yet, few studies have looked into such disparities in drought-induced undernutrition, possibly due to the limitations of traditional methods of identifying effect modifiers.
